# Bevacizumab and sunitinib mediate osteogenic and pro-inflammatory molecular changes in primary human alveolar osteoblasts in vitro

**DOI:** 10.1007/s10266-022-00691-y

**Published:** 2022-02-16

**Authors:** Elena Hofmann, Benedikt Eggers, Nils Heim, Franz-Josef Kramer, Marjan Nokhbehsaim, Werner Götz

**Affiliations:** 1 Department of Oral, Maxillofacial and Plastic Surgery, University Hospital Bonn, Welschnonnenstr 17, 53111 Bonn, Germany; 2 Department of Oral and Maxillofacial Surgery, Charité- Universitätsmedizin Berlin, Corporate member of Freie Universität Berlin, Humboldt-Universität Zu Berlin, and Berlin Institute of Health, Augustenburger Platz 1, 13353 Berlin, Germany; 3 Section of Experimental Dento-Maxillo-Facial Medicine, University Hospital Bonn, 53111 Bonn, Germany; 4 Department of Orthodontics, University Hospital Bonn, 53111 Bonn, Germany

**Keywords:** MRONJ, Alveolar osteoblasts, Bevacizumab, Sunitinib

## Abstract

Antiangiogenic medications target the de novo blood vessel formation in tumorigenesis. However, these novel drugs have been linked to the onset of medication-related osteonecrosis of the jaw (MRONJ). The aim of this in vitro study was to examine the effects of the vascular endothelial growth factor A (VEGFA) antibody bevacizumab (BEV) and the receptor tyrosine kinase inhibitor (RTKI) sunitinib (SUN) on primary human osteoblasts derived from the alveolar bone. Primary human alveolar osteoblasts (HAOBs) were treated with BEV or SUN for 48 h. Cellular metabolic activity was examined by XTT assay. Differentially regulated genes were identified by screening of 22 selected osteogenic and angiogenic markers by quantitative real-time reverse transcriptase polymerase chain reaction (qRT^2^-PCR). Protein levels of alkaline phosphatase (ALP), collagen type 1, α1 (COL1A1) and secreted protein acidic and cysteine rich (SPARC) were examined by enzyme-linked immunoassay (ELISA). Treatment with BEV and SUN did not exhibit direct cytotoxic effects in HAOBs as confirmed by XTT assay. Of the 22 genes examined by qRT^2^-PCR, four genes were significantly regulated after BEV treatment and eight genes in the SUN group as compared to the control group. Gene expression levels of ALPL, COL1A1 and SPARC were significantly downregulated by both drugs. Further analysis by ELISA indicated the downregulation of protein levels of ALP, COL1A1 and SPARC in the BEV and SUN groups. The effects of BEV and SUN in HAOBs may be mediated by alterations to osteogenic and catabolic markers. Therapeutic or preventive strategies in MRONJ may address drug-induced depression of osteoblast differentiation.

## Introduction

The development of antiangiogenic medications that target the deprivation of tumor vasculature and blood supply has led to promising treatment strategies in oncology as well as ophthalmological vascular-related diseases [1–4]. Yet, antiangiogenic agents have been linked to the occurrence of medication-related osteonecrosis of the jaw (MRONJ) [5].

MRONJ is characterized by the progressive destruction of alveolar bone, which is associated with significant morbidity [6]. The clinical picture of MRONJ is an alveolar bone exposed to the oral cavity or bone that can be probed through an intraoral or extraoral fistula in the maxillofacial region persisting for more than 8 weeks and current or previous treatment with antiresorptive or antiangiogenic agents without a history of radiation therapy to the craniofacial region or obvious metastatic disease to the jaws [6]. Progression of the disease can involve loss of teeth, necrosis of adjacent regions and pathologic fractures. Dental extraction (61.7%) and oral surgery (7.2%) were identified as major risk factors [7]. MRONJ is predominantly found in areas with thin mucosa overlying bone prominences and located more frequently in the mandible than in the maxilla [8, 9].

While necrotic bone may remain asymptomatic for years, symptoms of MRONJ are pain, tooth mobility, suppuration, inflammation of the oral mucosa, visible ulceration or bone sequestration. The complex treatment regimen includes surgical interventions, antibiotic treatment and/or prolonged inpatient stays [6, 10]. In 2003, Marx was the first to report the occurrence of necrotic lesions of the jaw after bisphosphonate (BP) therapy [11]. Today, MRONJ is considered a serious complication experienced by some patients receiving treatment for malignancies, osteoporosis or other skeletal-related diseases. The associated agents exert antiresorptive or antiangiogenic functions and include BPs, e. g. zoledronate and pamidronate, as well as denosumab, a direct inhibitor of the receptor activator of NF-κB ligand (RANKL). More recently, the vascular endothelial growth factor A (VEGFA)-neutralizing antibody bevacizumab (BEV) and the receptor tyrosine kinase inhibitor (RTKI) sunitinib (SUN) have been associated with MRONJ [5]. In addition, novel drugs have been linked to the onset of MRONJ, for example the kinase inhibitors axitinib, imatinib and sorafenib [5]. The International Task Force on MRONJ reports a 1–15% incidence in oncological patients receiving antiresorptive agents, such as BPs or denosumab [12]. The risk of MRONJ is higher after combined BP and anti-VEGFA therapy with an incidence of up to 2.4% than after antiangiogenic monotherapy with an incidence of up to 0.2% [13].

BEV is a monoclonal antibody designed to neutralize the biological activity of VEGFA by inhibiting its attachment to VEGFR1 and VEGFR2 [14, 15]. Sunitinib malate (SU-11248/ Sutent^®^; Pfizer Pharma, Berlin, Germany) is an oral RTKI targeted at VEGFR1, VEGFR2, VEGFR3 and platelet-derived growth factor receptors (PDGFR)α/β amongst others [16, 17]. In addition to other indications, BEV is applied in the treatment of metastatic colorectal cancer (mCRC) and metastatic renal cell carcinoma (mRCC) [3, 4]. The indications of SUN comprise the treatment of mRCC, gastrointestinal stromal tumor (GIST) and other oncological diseases [2].

The pathophysiology of MRONJ remains a source of great discussion and there are no definitive prevention and treatment strategies to date [10]. Evidence has accumulated that MRONJ is a multifactorial disease [6, 12, 18]. The hypothesized pathogenic mechanisms that have reached broad acceptance are based on inflammation and/or infection, antiangiogenic properties, decreased bone turnover, altered immunity and soft tissue toxicity of bone-modifying agents [18]. Previous studies have addressed the effects of BPs in oral squamous cell carcinoma cultures [19] or murine osteoblast precursor cells [20]. However, the effects of antiangiogenic agents on osteoblasts and osteoclasts derived from the jaw bone as the main cells involved in bone homeostasis are elusive.

Research suggests that multiple aspects contribute to the susceptibility of the jaw bone to the development of MRONJ [18]. The jaw bone experiences unique stress factors, including high occlusal forces, dental procedures, periodontal disease and microfractures. Unlike any other bone, the alveolar bone is only protected by a thin mucoperiostal layer and intimately connected to the oral microbiome via the periodontium [21]. Alterations to the signaling pathway dedicated to the osseous differentiation in neural crest cell-derived craniofacial bone may explain the site-specificity of drug-related osteonecrosis. It was found that BP treatment affects the signaling pathway of bone morphogenic protein (BMP) 2 in human jaw bone samples [22]. Higher BP concentrations in the jaw bone are associated with greater mineral content and higher bone turnover [23] and the site-specific formation of osteonecrosis [24]. When compared to long bones, the alveolar bone exhibits differences in matrix composition, BMP expression patterns, osteoclastic bone degradation and the mesenchymal stem cell (MSC) pool [25, 26]. Alveolar osteoblasts have a greater proliferation rate and osteogenic potential than long bone osteoblasts, thus accounting for the higher remodeling activity of alveolar bone [27, 28]. Furthermore, previous studies found the dysregulation of jaw bone homeostasis by BPs [29, 30]. While zoledronate impaired bone remodeling at dental extraction sites of the alveolar bone in mice and rats, tibia and ilium defects healed completely [24, 31].

Despite evidence of distinct characteristics of the jaw bone, only few studies investigate drug-mediated effects in osteoblasts derived from the alveolar bone [32, 33]. In addition, studies predominantly address antiresorptive medications, such as BPs as the first drugs linked to the onset of MRONJ [32, 34]. BPs inhibit osteoclast differentiation and function and increase apoptosis, subsequently leading to decreased bone remodeling [35]. Moreover, BPs may exhibit antiangiogenic properties that impair bone regeneration capacity [18]. Another study reports that increased sequestration of BPs in the jaws reduces blood supply and suppresses bone turnover, leading to site-specific necrotic lesions [8]. Soft tissue toxicity is also implicated in the pathophysiology of MRONJ after demonstrating antiproliferative and apoptotic effects of BPs in various cell types, including oral epithelial cells [36]. Research suggests that disrupted wound healing, inflammatory processes and the oral microbiome are involved in the development of MRONJ [9, 37]. Alterations to angiogenesis and the immune response by antiresorptive or antiangiogenic drugs may have detrimental effects on wound healing function [8, 38, 39].

The question of how the more recently introduced entity of antiangiogenic drugs such as BEV and SUN affects primary osteoblasts demands further investigation. Since BEV and SUN are commonly used in cancer therapy and both target VEGFA [14–17], we aimed to investigate similarities and differences in gene and protein expression changes. We tested the hypotheses that BEV and SUN decrease survival of primary human alveolar osteoblasts (HAOBs), inhibit angiogenesis, suppress bone remodeling and modulate cytokine expression [18]. To examine the effects of antiangiogenic agents on bone metabolism, important markers of osteoblast differentiation and remodeling were studied. In addition, growth factors and markers of angiogenesis were addressed, as both drugs exert antiangiogenic properties. The expression of pro-inflammatory cytokines and chemokines involved in wound healing was also analyzed. This study aims to elucidate the in vitro effects of BEV and SUN on angiogenic and osteogenic markers in HAOBs derived from the jaw bone.

## Materials and methods

### Isolation and culture of primary HAOBs

Primary HAOBs were derived by explant cultures from bone fragments discarded during routine teeth extractions or orthognathic surgery from healthy donors (aged 18–50 years) at the Department of Oral, Maxillofacial and Plastic Surgery at the University Hospital Bonn, according to the previously described protocol by Marolt et al. [40]. Informed consent was obtained from all patients and ethical approval was provided by the ethics committee of the University of Bonn (086/11). HAOBs were cultured in 75 cm^2^ culture flasks (Greiner bio-one GmbH, Frickenhausen, Germany) in Dulbecco’s modified Eagle medium (DMEM) supplemented with 10% fetal bovine serum (FBS) and 1% penicillin/streptomycin (P/S) (Life Technologies Corporation, Grand Island, USA) and maintained in a humidified incubator at 37 °C and 5% CO_2_.

### Characterization of osteoblasts

HAOBs at passages 3–5 were cultured at a density of 8000 cells/cm^2^ on cover slips placed in 6-well plates in osteogenic medium (DMEM supplemented with 1% FBS, 1% P/S, 10 nM dexamethasone, 280 µM ascorbic acid and 5 mM β-glycerophosphate (Sigma-Aldrich Chemie GmbH, Merck KgaA, Munich, Germany)). The medium was refreshed twice a week. After 28 days, cells were fixated using 4% paraformaldehyde and 0.05% Triton^®^ X-100 (all reagents obtained from Merck KgaA, Darmstadt, Germany).

For Alizarin red S staining, 270 µl 2% alizarin solution was added for 20 min at room temperature (RT), followed by 5 washing cycles with ddH_2_O. For von Kossa staining, cells were incubated with 500 µl of 5% silver nitrate solution for 40 min at 4 °C. After that, cells were washed twice using ddH_2_O before 500 µl of 1% pyrogallol was added for 5 min. Washing with ddH_2_O was repeated twice. Surplus silver was removed and the precipitates fixated by incubation with 500 µl sodium thiosulfate for 5 min. Finally, cells were washed twice with ddH_2_O and incubated with 500 µl 0.1% nuclear fast red-aluminum sulfate solution for 10 min to permit nuclear staining.

For immunocytochemistry (ICC), cells were blocked with 5% bovine serum albumin fraction V (Roche Diagnostics, Indianapolis, USA) in 1 × PBS for 40 min at RT. Serum block was removed and the primary antibodies of monoclonal anti-collagen I antibody [EPR7785] ab138492 (mouse, TaKaRa Bio Europe S.A.S., Saint-Germain-en-Laye, France) and monoclonal anti-bovine osteocalcin antibody M041 (mouse, TaKaRa Bio Europe S.A.S., Saint-Germain-en-Laye, France) were incubated for 1 h at RT. After three rinsing steps with 1 × PBS, Dako EnVision™ + system-HRP labelled polymer anti-mouse or anti-rabbit secondary antibody (Dako North America Inc., Carpinteria, USA) was added for 30 min at RT. After washing with 1 × PBS, staining was visualized with 10% DAB in 1 × stable peroxide substrate buffer. Cells were analyzed by light microscopy (Axioskop 2, Axiocam MRc, Axiovision 4.7/AutMess, Carl Zeiss Microscopy GmbH, Jena, Germany).

### BEV and SUN treatment

BEV (Avastin^®^; 100 mg/4 ml; Roche Pharma AG, Grenzach-Whylen, Germany) was diluted in starvation medium (supplemented with 1% FBS) to concentrations of 50 µg/ml, 150 µg/ml and 300 µg/ml on the day of the stimulation, based on doses described in the literature [41, 42]. SUN malate (Sutent^®^; 25 mg; Pfizer Pharma GmbH, Berlin, Germany) was solubilized in dimethyl sulfoxide (DMSO) (Sigma-Aldrich Chemie GmbH, Merck KgaA, Munich, Germany) to achieve a 100 µM SUN stock solution. On the day of the experiments, the stock solution was diluted in starvation medium to obtain working solutions of 40 nM, 200 nM, 500 nM and 1000 nM at a DMSO concentration of below 0.01% to exclude DMSO effects on cell cytotoxicity [43]. For the following experiments, HAOBs at passages 3–5 were used, seeded at a density of 10,000 cells per cm^2^ in growth medium two days prior to the initiation of treatment. Cells were synchronized in starvation medium supplemented with 1% FBS 24 h prior to the experiment to reduce serum effects. The next day, BEV and SUN treatment for 48 h was commenced.

### XTT proliferation assay

Cellular metabolic activity was quantified by XTT assay (Cell Proliferation Kit XTT, PanReac AppliChem, Darmstadt, Germany) to test for cytotoxic effects. HAOBs were treated with selected doses of BEV and SUN for 48 h in 96-well plates in technical sixfold replicates. The XTT reaction mixture was freshly prepared according to the manufacturer’s instruction and 50 µl per well containing 100 µl of sample was added with incubation for 4 h. Fluorescence was measured with a microplate reader at 475 nM and 660 nM absorbance. Background-subtracted absorbance values were normalized to the control group.

### Gene expression analysis using quantitative real-time polymerase chain reaction

To identify differently regulated genes following treatment with BEV and SUN, changes to expression levels of 23 selected genes were quantified with the use of qRT^2^-PCR. For RNA extraction, HAOBs were seeded in 6-well plates in technical triplicates. After treatment with selected doses of BEV and SUN for indicated culture periods, RNA was isolated using the RNeasy^®^ mini kit (Qiagen GmbH, Hilden, Germany) according to the manufacturer's instructions. The purity and concentration of 1 µl of RNA was photometrically measured using NanoDrop ND-2000. RNA samples were stored at − 80 °C. Total RNA was converted to cDNA by reverse transcriptase using iScript™ select cDNA synthesis kit (Bio-Rad Laboratories, Inc., Hercules, USA) according to the manufacturer’s manual. SYBR green-based qRT^2^-PCR was performed using QuantiTect primer assays of alkaline phosphatase (ALPL), bone gamma-carboxyglutamate (gla) protein (BGLAP), collagen type 1, α1 (COL1A1), matrix metalloproteinase (MMP)1, secreted phosphoprotein (SPP)1, secreted protein acidic and cysteine rich (SPARC), runt-related transcription factor (RUNX)2, Sp7 transcription factor (SP7), bone morphogenic protein (BMP)2, fibroblast growth factor (FGF)1, insulin-like growth factor (IGF)1, platelet-derived growth factor β polypeptide (PDGFB), tumor growth factor β (TGFB), angiopoietin (ANGPT)1, vascular endothelial growth factor (VEGF)A, vascular endothelial growth factor receptor (VEGFR)2, chemokine (C–C motif) ligand (CCL)2, cyclooxygenase (COX)2, interleukin (IL)1β (IL1B), IL6, IL8, tumor necrosis factor α (TNFA) and glyceraldehyde 3-phosphate dehydrogenase (GAPDH) (Qiagen GmbH, Hilden, Germany).

### Enzyme-linked immunoabsorbance assay

Cells were seeded in 6-well plates in technical triplicates for ELISA sample collection. Protein levels of key factors involved in osteogenesis, ALP, COL1A1 and SPARC in the supernatants of untreated and treated HAOBs were analyzed by commercially available detection kits ((human ALP ELISA (RayBiotech Life, Inc., Peachtree Corners, USA); human Pro-Collagen I α1 ELISA (R&D Systems Inc., Minneapolis, USA); human SPARC/Osteonectin ELISA (RayBiotech Life, Inc., Peachtree Corners, USA)) according to the manufacturer’s instructions. The protein content of each sample was determined using the BCA Protein Assay kit (Thermo Fisher Scientific Inc., Waltham, USA). Absorbance was measured with a microplate reader (Epoch™ Microplate Spectrophotometer, BioTek Instruments, Inc., Winooski, USA) at 450 nM.

### Statistical analysis

Statistical analysis was performed and graphs were created using GraphPad Prism Software Version 8 (GraphPad Software, Inc., La Jolla, California, USA). Testing of normal distribution was conducted by Shapiro–Wilk normality test. Moreover, one-way analysis of variance (ANOVA), unpaired *t* tests and correction for multiple comparisons using the Holm–Šídák method were performed. *P* values below 0.05 were defined as statistically significant. Results display mean values with the standard error of the mean (SEM). Asterisks represent the level of statistical significance: * = *p* < 0.05, ** = *p* < 0.01 and *** = *p* < 0.001.

## Results

### Osteoblast phenotype confirmation

**Fig. 1:**
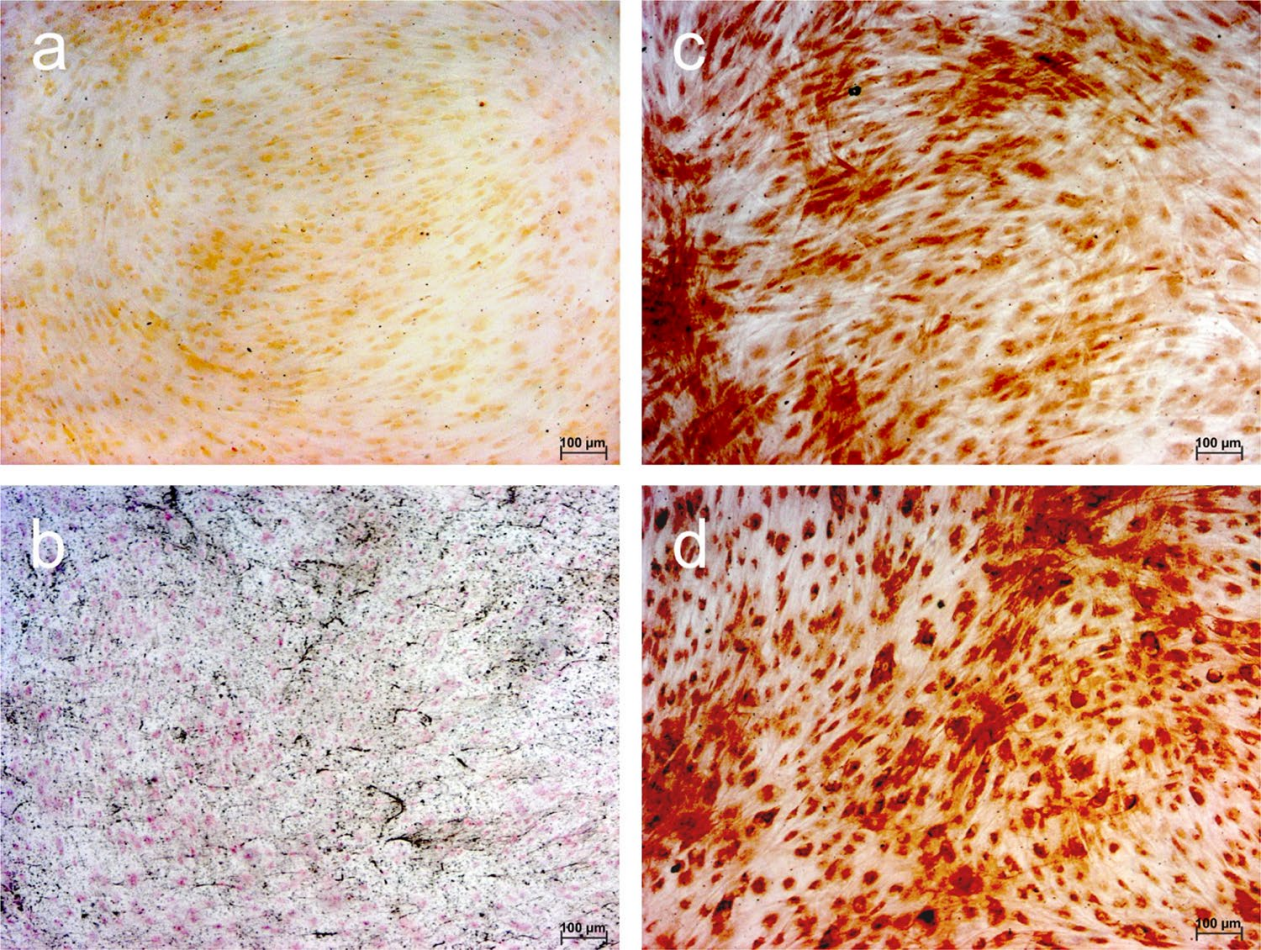
Confirmation of osteoblast phenotype by histochemical staining using alizarin and von Kossa and expression of osteocalcin and type I collagen. Positive mineralization was confirmed by (**a**) alizarin red S and (**b**) von Kossa staining after 28 days of culture in osteogenic medium (×10 magnification, scale bar = 100 µm). ICC demonstrated protein expression of (**c**) osteocalcin and (**d**) type I collagen (×10 magnification, scale bar = 100 µm)

Primary cells pooled from alveolar bone fragments stained positively for the presence of calcium (alizarin red S, Fig. 1a) and calcium phosphate (von Kossa, Fig. 1b) indicating their mineralization when cultured in osteogenic medium. ICC confirmed protein expression of osteocalcin and type I collagen (Fig. 1 c, d). Furthermore, HAOBs expressed osteogenic markers during qRT^2^-PCR (Fig. 3).

### Effects of BEV and SUN on the metabolic activity of HAOBs

**Fig. 2:**
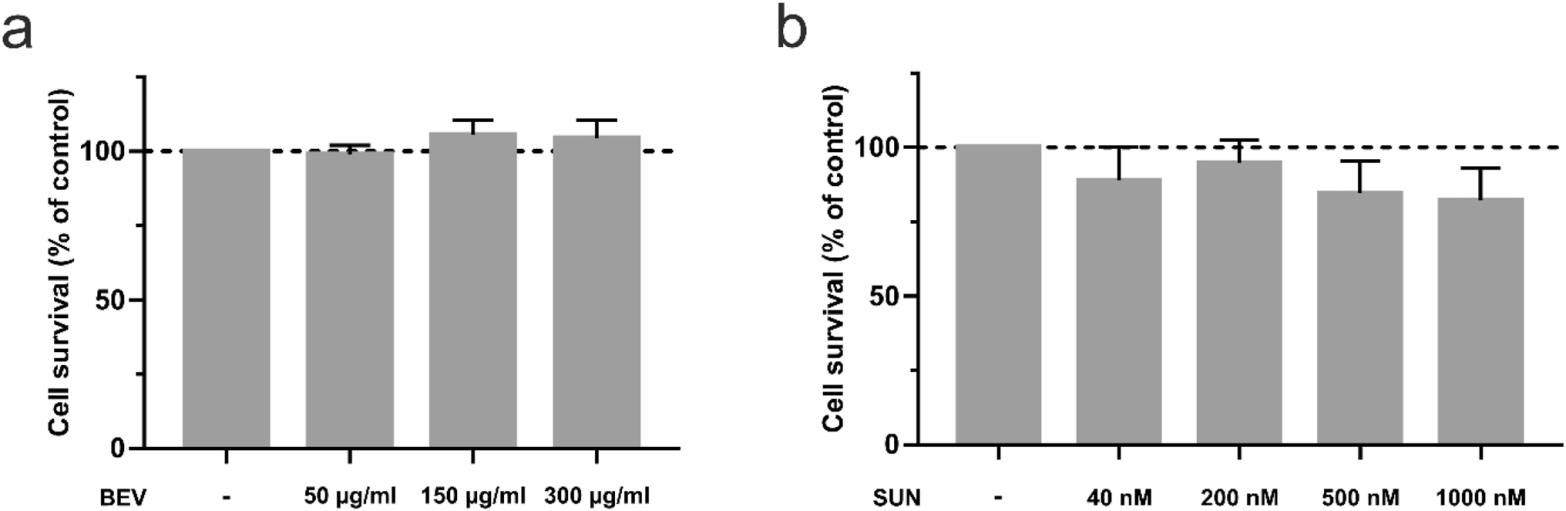
Effects of BEV and SUN on cell proliferation in HAOBs. HAOBs were treated with respective doses of (**a**) BEV and (**b**) SUN in starvation medium for 48 h and cellular metabolic activity was determined by XTT assay. The control group (-) received starvation medium. Five independent experiments were performed with six technical replicates each (*n* = 5). Data are expressed as a percentage of the untreated control and represent mean ± SEM (one-way ANOVA, not significant). Dashed line represents control levels set at 100%

A XTT assay served to exclude direct cytotoxic effects of the selected doses of BEV and SUN in HAOBs. No differences between untreated and treated HAOBs could be detected (Fig. 2) and a direct cytotoxic effect was rejected.

### Relative qRT^2^-PCR expression of osteogenic, angiogenic and pro-inflammatory genes

**Fig. 3:**
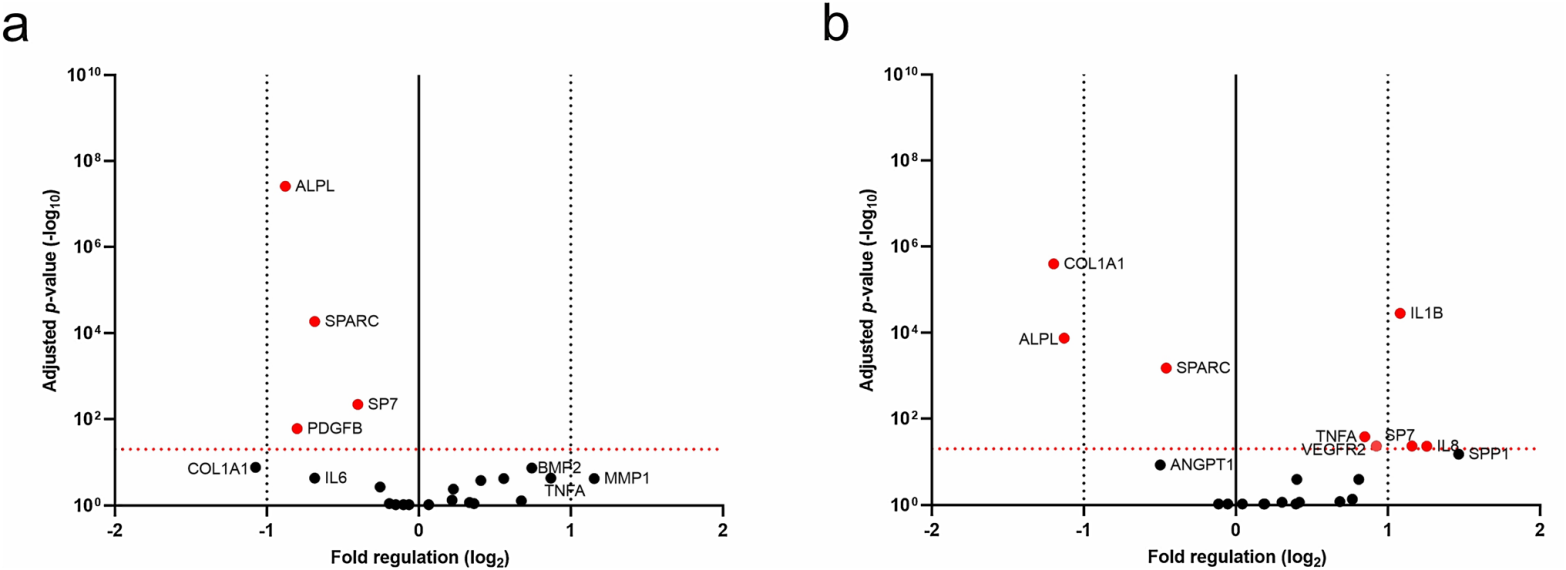
Volcano plots illustrating the expression levels of 22 genes in HAOBs after treatment with BEV and SUN. HAOBs were treated with (**a**) 300 µg/ml BEV and (**b**) 1000 nM SUN in starvation medium for 48 h (*n* = 5). qRT^2^-PCR was performed to identify regulated genes as compared with the control group that received starvation medium. The log_2_ of the expression levels in relation to the expression levels of the house keeping gene GAPDH (fold change; 2^−∆∆Cq^) are plotted against the log_10_ of the adjusted *p* values for the treatment versus the control group. An adjusted *p* value below 0.05 was considered as statistically significant and represented by the horizontal dotted red line. The vertical solid line displays no change in fold regulation and the vertical black dotted line represents a fold change of ± 2. Labeled red dots display significantly regulated genes (*p* < 0.05)

To test for the induction of an inflammatory state or the alteration of the cellular metabolism by BEV and SUN, characteristic chemokines and cytokines, as well as angiogenic markers, extracellular matrix (ECM) and differentiation markers were studied. Gene expression levels in HAOBs were analyzed by qRT^2^-PCR to test for a dysregulation of bone physiology by anti-VEGFA agents. Volcano plots were compiled to identify genes regulated after 48 h of treatment with 300 µg/ml BEV and 1000 nM SUN. Of the 22 genes tested in HAOBs, four genes were significantly regulated after BEV treatment, while eight genes were regulated in the SUN group as compared to the control group (Fig. 3, Table 1).

**Table 1:** Comparison of regulated genes after BEV and SUN treatment

Genes	Gene abbrev	BEV/control			SUN/control		
		Mean FR	*P* value	Adjusted *p* value	Mean FR	*P* value	Adjusted *p* value
Extracellular matrix (ECM) molecules							
Alkaline phosphatase	ALPL	**0.5**	** < 0.001**	** < 0.001**	**0.5**	** < 0.001**	** < 0.001**
Bone gamma-carboxyglutamate (gla) protein	BGLAP	1.2	0.143	0.750	1.7	0.122	0.728
Collagen type 1, α1	COL1A1	0.5	0.008	0.131	**0.4**	** < 0.001**	** < 0.001**
Matrix metalloproteinase 1	MMP1	2.2	0.021	0.237	1.8	0.024	0.255
Secreted phosphoprotein 1	SPP1	1.3	0.238	0.851	2.8	0.005	0.066
Secreted protein acidic and cysteine rich	SPARC	**0.6**	** < 0.001**	** < 0.001**	**0.7**	** < 0.001**	** < 0.001**
Skeletal development							
Runt-related transcription factor 2	RUNX2	0.9	0.319	0.901	1.3	0.378	0.942
Sp7 transcription factor	SP7	**0.8**	** < 0.001**	**0.005**	**2.2**	**0.003**	**0.043**
Cell growth and differentiation							
Bone morphogenic protein 2	BMP2	1.7	0.009	0.136	1.2	0.216	0.857
Fibroblast growth factor 1	FGF1	0.9	0.525	0.949	1.0	0.844	0.942
Insulin-like growth factor 1	IGF1	1.5	0.019	0.237	1.1	0.388	0.942
Platelet-derived growth factor β polypeptide	PDGFB	**0.6**	**0.001**	**0.017**	1.1	0.481	0.942
Tumor growth factor β	TGFB	1.3	0.025	0.264	1.0	0.735	0.942
Angiogenic growth factors							
Angiopoietin 1	ANGPT1	1.0	0.619	0.949	0.7	0.010	0.118
Vascular endothelial growth factor A	VEGFA	1.0	0.655	0.949	0.9	0.403	0.942
Vascular endothelial growth factor receptor 2	VEGFR2	0.8	0.041	0.372	**1.9**	**0.003**	**0.043**
Cytokines and chemokines							
Chemokine (C–C motif) ligand 2	CCL2	1.3	0.351	0.901	1.6	0.179	0.830
Cyclooxygenase 2	COX2	1.2	0.052	0.415	1.3	0.242	0.857
Interleukin 1β	IL1B	1.6	0.169	0.773	**2.1**	** < 0.001**	** < 0.001**
Interleukin 6	IL6	0.6	0.017	0.233	1.3	0.024	0.255
Interleukin 8	IL8	0.9	0.541	0.949	**2.4**	**0.003**	**0.044**
Tumor necrosis factor α	TNFA	1.8	0.016	0.233	**1.8**	**0.001**	**0.026**

Cells with values in bold indicate significant genes

*FR* = *fold regulation*

Gene expression changes to the osteogenic markers ALPL, BGLAP, COL1A1, MMP1, SPP1 and SPARC were investigated. After treatment with BEV, the expression levels of ALPL (*p* < 0.001) and SPARC (*p* < 0.001) were significantly downregulated. Similarly, ALPL, COL1A1 and SPARC were significantly decreased in the SUN treatment group (*p* < 0.001, respectively). The expression of MMP1, an essential regulator of bone mass and the ECM turnover [44], was upregulated by BEV (*p* = 0.237) and SUN (*p* = 0.255). Furthermore, SPP1 expression was upregulated in BEV- (*p* = 0.851) and SUN-treated cells (﻿p = 0.066).

Reduced gene expression of osteogenic markers and factors involved in the skeletal development prompted the investigation of the critical transcription factors of osteoblast differentiation RUNX2, also known as core binding factor α 1 (CBFA1) and osterix, a zinc finger-containing protein, also named SP7 [45] to assess alterations to bone remodeling. SP7 was significantly downregulated after treatment with BEV (*p* = 0.005) but upregulated after treatment with SUN (*p* = 0.043).

Osteoblast differentiation is induced by BMPs, in particular BMP2 and BMP4, TGFβ, PDGF, IGF and FGF among others [46, 47]. We tested the effects of BEV and SUN on the gene expression of growth factors that are intimately related to angiogenesis and osteogenesis, including BMP2, FGF1, IGF1, PDGFB and TGFB. PDGFB expression was significantly reduced in the BEV treatment group (*p* = 0.017). Since both agents have antiangiogenic properties, the angiogenic markers ANGPT1, VEGFA and VEGFR2 were also investigated. Treatment with SUN led to the significant upregulation of VEGFR2 (*p* = 0.043).

Prolonged activation of pro-inflammatory cytokines after antiresorptive and antiangiogenic drugs may contribute to impaired wound healing and bone repair and play a role in the development of osteonecrotic lesions [9, 37]. Hence, the gene expression of CCL2, COX2, IL1B, IL6, IL8 and TNFA was analyzed. The pro-inflammatory cytokines IL1B (*p* < 0.001), IL8 (*p* = 0.044) and TNFA (*p* = 0.026) were significantly increased in the SUN treatment group.

### Effects of BEV and SUN on ALPL, COL1A1 and SPARC gene expression

**Fig. 4:**
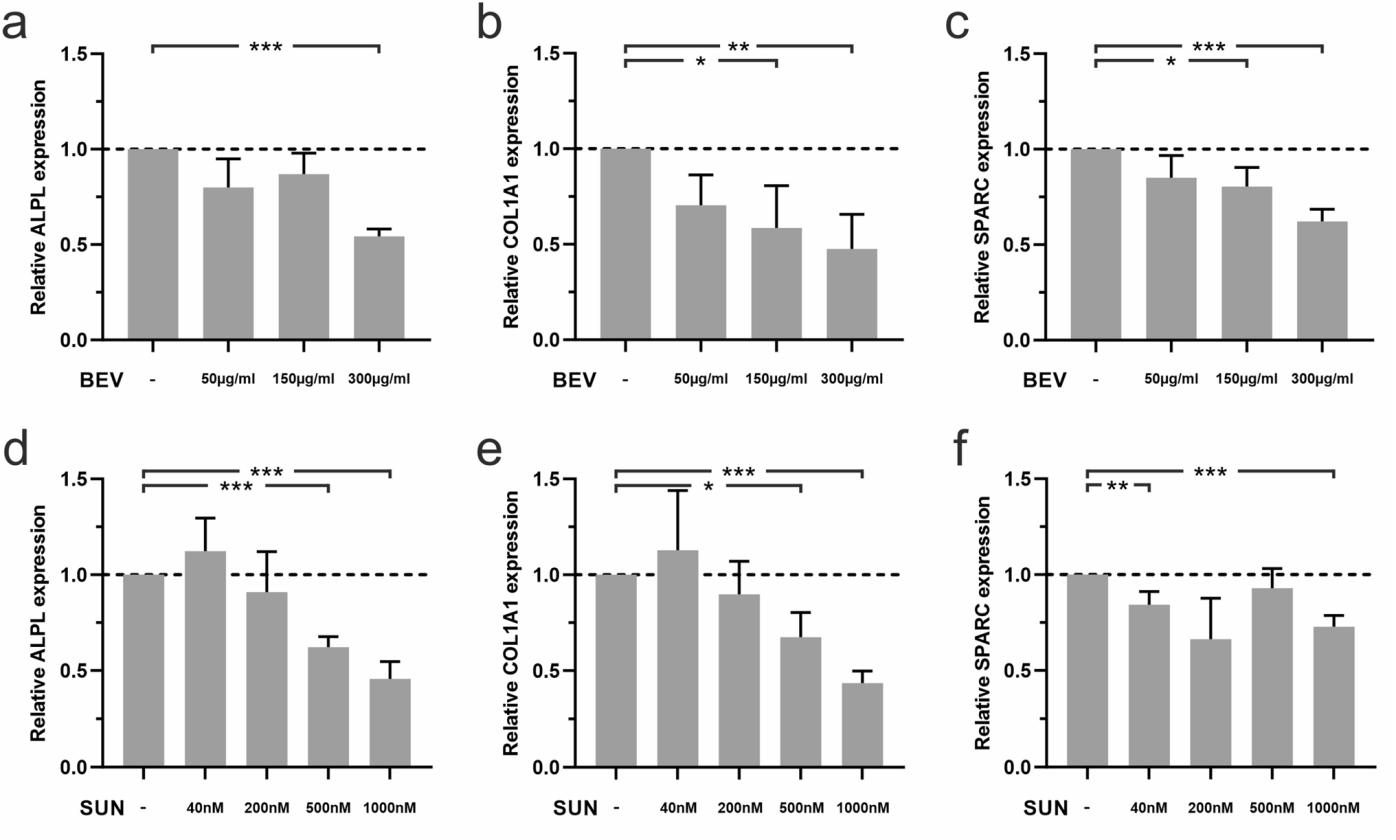
Effects of BEV and SUN treatment on relative gene expression levels of ALPL, COL1A1 and SPARC in HAOBs. HAOBs were treated with respective doses of (**a**–**c**) BEV and (**d**–**f**) SUN in starvation medium for 48 h. The control group (-) received starvation medium. qRT^2^-PCR was performed. Gene expression levels were calculated in relation to the control, which was set at the level of one. Independent experiments were performed in technical triplicates (*n* = 5). Data are mean ± SEM (unpaired *t* test, * = *p* < 0.05, ** = *p* < 0.01, *** = *p* < 0.001). Dashed line represents control levels set at 1

The effects of BEV and SUN treatment on HAOBs were elucidated by gene expression analyses of the osteoblast differentiation markers ALPL, COL1A1 and SPARC using qRT^2^-PCR. BEV- and SUN-treated HAOBs showed a strong tendency towards a concentration-dependent downregulation of ALPL, COL1A1 and SPARC after 48 h of treatment (Fig. 4). Expression levels at 300 µg/ml BEV compared to the control were significantly reduced for ALPL (*p* < 0.001), COL1A1 (*p* < 0.01) and SPARC (*p* < 0.001) (Fig. 4a–c). In addition, the downregulation of COL1A1 expression was statistically significant in the 150 µg/ml BEV group. Similarly, ALPL, COL1A1 and SPARC expression levels were significantly decreased at 1000 nM SUN (*p* < 0.001, respectively) (Fig. 4d–f). Furthermore, ALPL expression and COL1A1 expression were significantly reduced at 500 nM SUN (*p* < 0.001 and *p* < 0.05, respectively). The decrease in SPARC expression was statistically significant at 40 nM (*p* < 0.01) in SUN-treated HAOBs. In synopsis, the two tested agents significantly decreased the gene expression levels of ALPL, COL1A1 and SPARC that play an important role in osteoblast differentiation and bone mineralization.

### Effects of BEV and SUN on the protein expression of ALP, COL1A1 and SPARC

**Fig. 5:**
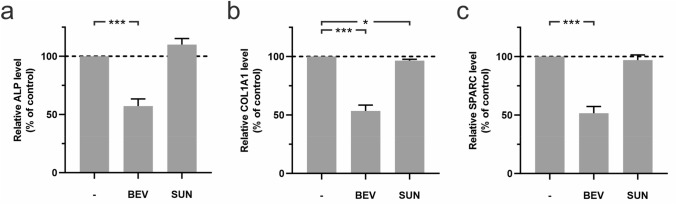
Effects of BEV and SUN treatment on protein expression levels of COL1A1, ALPL and SPARC in HAOBs. HAOBs were treated with 300 µg/ml BEV and 1000 nM SUN in starvation medium for 48 h (*n* = 5). The control group (-) received starvation medium. Supernatants of HAOBs were collected and ELISA was performed to determine protein secretion of (**a**) ALP, (**b**) COL1A1 and (**c**) SPARC by HAOBs following treatment with BEV and SUN. Protein expression levels were normalized to total protein content determined by BCA assay. Data represent expression levels relative to the control and are mean values ± SEM (unpaired *t* test, * = *p* < 0.05, *** = *p* < 0.001). Dashed line represents control levels set at 100%

Based on the findings that gene expression levels of ALPL, COL1A1 and SPARC were significantly downregulated after 48 h of treatment with BEV and SUN, we next wished to test for changes to protein expression levels in HAOBs by ELISA. Our results indicated the downregulation of ALP, COL1A1 and SPARC protein expression by HAOBs following treatment with antiangiogenic agents (Fig. 5**a**–**c**) with a stronger downregulation following treatment with BEV when compared to SUN. ALP, COL1A1 and SPARC expression levels were significantly downregulated by about 50% in the BEV group (*p* < 0.001, respectively), while COL1A1 expression was significantly reduced in the SUN group by about 4% (*p* < 0.05). Overall, treatment with BEV was associated with the downregulation of gene expression but also protein expression of ALP, COL1A1 and SPARC. In the SUN group, COL1A1 protein expression was significantly downregulated. Regulatory effects on ALP and SPARC protein expression by SUN were minor compared to BEV-treated cells.

## Discussion

MRONJ is a serious, debilitating condition associated with antiresorptive and antiangiogenic therapies. While anti-VEGFA agents have been extensively studied in endothelial and tumor cells, their effects on osteoblasts of the jaw bone that are most likely involved in the development of MRONJ remain elusive [18]. Elucidating the pathogenesis of MRONJ will aid in the quest for successful prevention and treatment strategies. The present study examined the in vitro effects of the VEGFA inhibitor BEV and the RTKI SUN on HAOBs in terms of cell behavior and gene expression.

To the best of our knowledge, this is the first study to examine the effects of BEV and SUN in primary human osteoblasts derived from the alveolar bone. The HAOB phenotype was confirmed immunocytochemically, functionally and by gene expression analysis. The present method using primary cells derived from the jaw bone most closely aligns with the cells affected by MRONJ. Furthermore, primary cells maintain physiological cell morphology and behavior, most likely mimicking in vivo characteristics rather than immortalized cell lines that have been cultured for decades and may have lost certain functional properties [48].

Previous in vitro studies reported antiproliferative and apoptotic effects at concentrations of 2 mg/ml for BEV [15] and 1 µM or more for SUN [49]. These, however, exceeded clinically relevant doses of 100–500 μg/ml for BEV [50, 51] and 50–100 ng/ml for SUN [17, 52], respectively. When used at clinically relevant doses BEV treatment exhibited antiangiogenic effects that reduced tumor growth in an animal neuroblastoma model [51]. Similarly, SUN reduced cell growth in RCC cell lines via an antiangiogenic mechanism instead of direct antitumor effects [52]. In line with previous research, the drug concentrations employed in this study exhibited no direct cytotoxic effects in HAOBs in the XTT assay. The present results indicated that direct cytotoxic effects on HAOBs in the development of MRONJ appear unlikely.

The intimate relation between angiogenesis and osteogenesis occupies a critical role in bone formation, remodeling and regeneration. Vascular supply is essential for the delivery of oxygen and nutrients for skeletal maintenance and bone cell differentiation [53]. Moreover, bone cells secrete factors dedicated to angiogenesis [54]. Using qRT^2^-PCR, 22 angiogenic and osteogenic genes were tested for the regulation by the anti-VEGFA agents during a culture period of 48 h. The present data showed the significant regulation of four genes in the BEV group and eight genes in the SUN group compared to the control group. ALPL and SPARC were significantly downregulated following treatment with BEV. PDGFB and SP7 expression levels were also significantly decreased. In the SUN group, ALPL, COL1A1 and SPARC gene expression levels were significantly decreased. Expression levels of SP7, VEGFR2, IL1B, IL8 and TNFA were significantly upregulated in SUN-treated cells. While the present data indicated that BEV and SUN generate similar effects in HAOBs, some genes were differently expressed after antiangiogenic treatment. In contrast to BEV, SUN may address multiple autocrine and paracrine signaling pathways [55].

This study found the significant dose-dependent downregulation of ALPL, COL1A1 and SPARC gene expression in HAOBs by BEV and SUN. The gene data results suggested that antiangiogenic treatment disrupts matrix formation and bone remodeling. In addition to these significant alterations to gene expression levels, protein expression levels of ALP, COL1A1 and SPARC by HAOBs were downregulated after treatment with BEV. Interestingly, SUN had only minor effects on the protein expression of ALP and SPARC. The expression of the COL1A1 protein was significantly downregulated in the SUN treatment group. The differences in protein expression between BEV- and SUN-treated cells may be due to the fact that SUN targets VEGFR1, VEGFR2, VEGFR3 and PDGFRα/β, which are downstream of the target of BEV [14–17]. This study, however, did not examine signaling pathways involved. Further research is needed to elucidate the specific signaling pathways of BEV and SUN. However, the current findings are supported by previous reports that demonstrated the significant downregulation of ALPL and COL1A1 gene expression after BP treatment [32]. Another study showed the decreased mineralization accompanied by reduced ALPL and osterix expression in osteosarcoma cells after treatment with the RTKI imatinib and nilotinib [56]. Zoledronic acid was found to decrease bone mineralization by directly inhibiting cell proliferation, osteoblast differentiation and osteoblast function of pre-osteoblastic cells and mesenchymal stem cells. RUNX2 and COL1A1 mRNA expression levels were downregulated [20]. Similarly, zoledronic acid treatment of osteoblasts decreased proliferation and osteogenic properties, while inducing pro-inflammatory marker expression [57].

This study further examined the regulation of RUNX2 and SP7 that are critical transcription factors of the skeletal development and required during osteoblast differentiation [45]. SP7 is a transcriptional regulator of the final stages of bone formation, but also mediates the commitment of MSCs to the osteoblastic lineage. SP7 expression was significantly downregulated by BEV, but significantly increased following SUN treatment. Since RUNX2 acts upstream of SP7 [45] and was not altered by antiangiogenic treatment in this study, effects of BEV and SUN on SP7 expression may be mediated via alternative signaling pathways. This concept is supported by the previously proposed RUNX2-independent signaling pathways for ossification [58]. Bone formation is disturbed in osterix-null mice despite normal levels of RUNX2 [45]. The downregulation of SP7 may contribute to decreased osteoblast differentiation and impaired bone remodeling, implicated in the pathophysiology of MRONJ. Opposing trends for SP7 expression suggest differential effects of BEV and SUN.

Previous research indicated an involvement of MMPs in the development of MRONJ [59]. MMP1 cleaves interstitial collagens and digests other ECM molecules and soluble proteins [44]. The results indicated increased gene expression levels of MMP1 by HAOBs following treatment with BEV and SUN. The induction of MMP1 gene expression may reflect bone matrix degradation and contribute to the development of MRONJ. It is possible that greater MMP1 regulation may be detectable in an animal model and following longer incubation periods. This should be a focus of future research. Elevated SPP1 expression may indicate an adaptive mechanism to maintain tissue homeostasis despite BEV and SUN treatment.

Growth factors, such as TGFB, are involved in wound healing, angiogenesis and bone regeneration [60]. Elevated BMP2, IGF1 and TGFB levels after antiangiogenic treatment may implicate disturbed tissue repair mechanisms that have been postulated in the pathogenesis of MRONJ. A previous study hypothesized a correlation between upregulated BMP2 and TGFB expression by BPs and an altered inflammatory response in HAOBs [32]. In contrast to the upregulated PDGFB expression after treatment with BPs [32], the study found the significant downregulation of PDGFB by BEV treatment. These findings suggest different molecular effects of BPs and anti-VEGFA agents. Amongst other factors, PDGFB induces osteoblast differentiation [46]. An altered PDGFB expression may account for suppressed osteoblast differentiation and the development of MRONJ.

Bone damage is associated with vascular disruption that leads to hypoxia. Hypoxia stimulates VEGFA synthesis and secretion to restore blood flow to the damaged site and subsequent osteogenesis [61]. The downregulation of ANGPT1 expression in the SUN group may indicate the dysregulation of angiogenesis. Similarly, absent effects on VEGFA expression after treatment with BEV and SUN may allude to defect bone repair mechanisms. Cell culture experiments in the osteoblast lineage successfully employ BEV and SUN to counter-regulate elevated VEGFA expression [62, 63].

A feedback mechanism in tumor cells may be activated by angiogenic inhibitors to restore VEGFA–VEGFR signaling [64]. Zoledronate was found to induce incorrect processing of VEGFR2 and aberrant VEGFR2 accumulation within vascular endothelial cells that could lead to the inhibition of chemotaxis towards VEGFA and disrupt angiogenesis [65]. In the present study, VEGFR2 expression was downregulated following BEV treatment, but significantly upregulated in SUN-treated HAOBs. These findings suggest distinctive patterns of VEGFR2 gene expression and may indicate divergent mechanistical pathways involved in MRONJ following BEV and SUN treatment.

During tissue repair, pro-inflammatory cytokines are secreted and inflammatory cells migrate to the wound site. The significant mRNA upregulation of inflammatory markers, such as IL1B, IL8 and TNFA, by SUN treatment may indicate the disruption of tissue repair. In contrast to SUN-treated HAOBs, there were no significant alterations to the gene expression levels of the tested cytokines and chemokines in the BEV treatment group, pointing to differences in gene expression changes by BEV and SUN. The study of the underlying signaling pathways could help to clarify these differences. A previous study demonstrated elevated cytokine expression levels in patients presenting with MRONJ following BP treatment with the greatest increase in IL1β expression [66]. Similarly, the pro-inflammatory potential was increased after zoledronic acid treatment in an animal model [67]. TNFα, IL1β and IL8 are amongst the osteoclastogenic cytokines that promote bone resorption [68], while TNFα and IL1 inhibit osteoblast differentiation and collagen synthesis [69]. Prolonged activation of pro-inflammatory cytokines after antiresorptive and antiangiogenic drugs may contribute to impaired wound healing and bone repair [37]. Reduced chemotaxis and disrupted osteoblast differentiation are reported following treatment with BPs, BEV and SUN [70–72]. Overall, the present results encourage previous research that hypothesized a role of persisting inflammation and compromised tissue repair by antiangiogenic agents in the development of osteonecrotic lesions [18, 37].

Osteoblasts regulate osteoclast differentiation and survival by RANKL expression [73]. In contrast to osteoblasts, multinucleated osteoclasts are derived from monocyte/macrophage precursors. The secretion of the decoy receptor osteoprotegerin of RANKL modulates osteoclast differentiation [74]. TNFα, IL1β and IL8 are amongst the cytokines that induce osteoclast differentiation, thus leading to bone resorption in the physiological bone remodeling cycle and in inflammatory diseases [68]. CXCL8 is similarly involved in osteoclastogenesis by promoting IL6 secretion by primary osteoblasts [75]. Pro-inflammatory cytokines create bone loss either by increasing osteoclast generation and activation or by inducing RANKL expression by osteoblasts [76, 77]. Another study found that TNFα, IL1 and IFNγ inhibit osteoblast differentiation and block collagen synthesis [69]. The association between disrupted bone remodeling and sustained inflammation marked by the increased secretion of pro-inflammatory and proresorptive cytokines is further encouraged by osteoporosis research [78].

Elucidating the pathogenesis of MRONJ may permit the identification of pharmacological targets and/or biomarkers in the prevention and treatment of MRONJ. On the other hand, current advances in antiangiogenic therapies aim at the improved targeting of anticancer drugs [1] and future research may be directed at the development of novel pharmacological agents with reduced risk of MRONJ. The ultimate goal is to limit the burden of MRONJ on patients, healthcare workers and health care systems in light of an increasing clinical use of antiangiogenic agents.

The present work suggests that the response of HAOBs to antiangiogenic agents involves multiple factors. BEV and SUN were found to regulate osteogenic gene expression, disrupt collagen synthesis and exhibit effects on the inflammatory response. The suppression of osteogenic markers may negatively affect bone homeostasis and contribute to the onset of osteonecrosis by antiangiogenic agents. After the successful implementation of MRONJ in an animal model [79], future in vivo studies may address the effects of antiangiogenic agents.
